# A migraine variant with abdominal colic and Alice in wonderland syndrome: a case report and review

**DOI:** 10.1186/1471-2377-10-2

**Published:** 2010-01-06

**Authors:** Sherifa A Hamed

**Affiliations:** 1 Department of Neurology and Psychiatry, Assiut University Hospital, Assiut, Egypt; 2 Dept. Integrative Systems Biology, George Washington University School of Medicine and Health Sciences, Research Center for Genetic Medicine, Children's National Medical Center, 111 Michigan Ave NW, Washington DC 20010, USA

## Abstract

**Background:**

Abdominal migraine is a commonly described migraine variant in children and young adults, but associations with Alice in Wonderland syndrome and lilliputian hallucinations are exceptional.

**Case presentation:**

A 20 years-old male experienced frequent and prolonged attacks of abdominal colic associated with autonomic manifestations started at the age of ten. At the age of 17, he additionally described prolonged attacks (≥ 7 days) of distortions of shape, size or position of objects or subjects. He said "Quite suddenly, objects appear small and distant (teliopsia) or large and close (peliopsia). I feel as I am getting shorter and smaller "shrinking" and also the size of persons are not longer than my index finger (a lilliputian proportion). Sometimes I see the blind in the window or the television getting up and down, or my leg or arm is swinging. I may hear the voices of people quite loud and close or faint and far. Occasionally, I experience attacks of migrainous headache associated with eye redness, flashes of lights and a feeling of giddiness. I am always conscious to the intangible changes in myself and my environment". There is a strong family history of common migraine. Clinical examination, brain-MRI and EEG were normal. Transcranial magnetic stimulation and evoked potentials revealed enhanced cortical excitability in multiple brain regions. Treatment with valproate resulted in marked improvement of all clinical and neurophysiological abnormalities.

**Conclusions:**

The association between the two migraine variants (abdominal migraine and Alice in Wonderland Syndrome) might have clinical, pathophysiological and management implications. I think this is the first description in the literature.

## Background

Migraine variants (MVs) or migraine equivalents exhibit themselves in forms other than head pain. MVs are less recognized and poorly understood entities of migraine. They are encountered in 3-5% of patients with migraine and usually affect children and young adults [1]. Abdominal migraine is a common MV. Typically, abdominal migraine affects ~20% of children with migraine. It occurs at the age of 5-9 years [2]. Also MVs may occur in the form of paroxysmal prolonged visual aura, paroxysmal vertigo, and rarely auditory, sensory or motor hallucinations. MVs with complex presentation are very rare; remain undefined by the International Classification of Headache Disorders (ICHD-II 2004) and seen in the literature as sporadic case reports [3-8]. Alice in Wonderland Syndrome (AWLS) is a rare MV. Collectively manifestations of AIWS include: micropsia (objects appear too small); macropsia (objects appear too big); teleopsia (objects seem too far away); lilliputianism (people appear too small); palinopsia (the persistence or recurrence of visual images after removal of the exciting stimulus); cerebral polyopia (the perception of multiple images); metamorphopsia (distortion of the shapes of objects); zoopsia (visual hallucinations containing complex objects, such as people and animals); achromatopsia (no perception of color); prosopagnosia (inability to recognize faces); visual agnosia (inability to recognize objects) and akinetopsia (loss of ability to perceive visual motion) [9]. Manifestations of AIWS were firstly described by Lippman [10]. In 1952, Lippman described seven migraineurs who had unusual distortions of body image. In 1955, Todd gave the syndrome its name [11]. Todd identified variety of self-experienced paroxysmal body schema disturbances as symptoms of AWLS (as depersonalization, derealization, visual illusions and disorders of the time perception). Historically, AWLS derived its name after Lewis Carroll book "Alice's Adventures in Wonderland". In the beginning of Carroll's book, the title character "Alice" found her-self growing and shrinking because of eating or drinking some strange creations. In fact, Lewis Carroll was said to have gotten migraine as early as 1856 and this helped him to inspire the fifth chapter of his book [12,13].

MVs should be differentiated from organic, psychiatric or central nervous system diseases with similar clinical presentation. In MVs, various molecular and cellular mechanisms may increase the susceptibility of cortical spreading depression (CSD), a main pathophysiologic mechanism associated with migraine. CSD is a slow cortical depolarization that progress from one brain area to its contiguous resulting in transient oligemia or ischemia, followed by hyperemia in different cortical areas and the associated multiple neurological phenomena [14].

## Case presentation

Twenty years-old Egyptian male was referred by an internist for neurological assessment. At presentation, the patient was curled up in a fetal position and only bent his head in response to questions, because he was experiencing intense and prolonged attacks of colic centered in his mid-abdomen. The colic attacks started at the age of ten. Early at the onset, the attacks were shorter in duration (few hours to 24 hours) and recurred every 3-5 months. They progressed in duration and frequency as in the last year before presentation, they were recurring weekly and each continued up to 72 hours. The attacks were accompanied by nausea, abdominal flushing, pallor, tachycardia and diarrhea (but never vomiting). The patient said that abdominal discomfort leaves as suddenly as it comes, offering no clue to when it will return. But he recognized eating fish or its products as strong triggers for the colic. The pain was severe enough to make him refuse to eat or drink for prolonged periods. This resulted in unintentional weight loss (the patient was 45 kilos at presentation), slowed growth and limited life activities and school attendance. The referral internist excluded the presence of organic disorder as a cause of patient's manifestations based on medical evaluation and unremarkable laboratory tests (blood, urine and stool analyses), abdominal imaging and endoscopic examinations. Long-term anti-epileptic treatment with carbamazepine was prescribed to the patient by a pediatrician based on the presence of epileptic activity in the electroencephalogram (EEG). Long-term treatment with colchicine was prescribed by another internist because of the misdiagnosis as Mediterranean Fever although the patient never experienced attacks of fever or serositis.

At the clinic and in front of the consulting neurologist, the patient developed the attacks of abdominal colic. The patient was not only feeling miserable, but looked it too. He was pale and curled up with sucked abdomen. Neither loss of consciousness nor postictal confusion was witnessed. Immediate EEG was normal.

At the age of 17, the patient developed abnormal visual phenomena in between the attacks of abdominal colic which lasted longer than a week. The patient said: "Quite suddenly everything seems strange, unusually I see objects small and distant (teliopsia) or large and close (peliopsia). I feel that I am getting shorter and smaller "shrinking" and also the size of persons are not longer than my index finger (a Lilliputian proportion). I see the same by either eye separately. I see the blind in the window or the television gets up and down, or my leg or arm is swinging. I hear people's voices loud and close or faint and far. I am always conscious of the intangible changes in myself and my environment. I am least likely to be distressed by those hallucinations as I find them non-threatening and I also get familiar with them. Occasionally at the end of the third day of an abdominal attack, I develop attacks of throbbing hemicranial headache associated with eye redness, flashes of lights and a feeling of giddiness which lasts no longer than 15 minutes". There is a strong family history (his father, 2 sisters and one brother) of recurrent hemicranial headaches associated with eye redness, anxiety, nausea and vomiting, that lasted for a period of hours to 3 days and sometimes improved with NSAIDs. The manifestations are suggestive of a common migraine disorder. There were no previous histories of trauma, drug ingestion, epilepsy, other central nervous system or psychiatric disorders.

Diagnostic testing was done to distinguish primary from secondary causes that share similar features. Diagnostic workup did not reveal another cause. MRI-brain (regular and diffusion-weighted) was normal. Interictal video EEG using basal temporal (T1/T2, T9/T10 and F9/F10) and sphenoidal electrodes did not reveal epileptiform abnormalities. For research purpose, sensory evoked potentials [SEPs: visual (VEP), brainstem auditory (BAEP) and somatosensory (SSEP) evoked potentials)] and transcranial magnetic stimulation (TMS) were done five times (before start of treatment and at the end of 15, 30, 60 and 90 days after treatment) to assess the excitability and function of the motor and sensory cortices. In each test of SEPs, a peripheral sense organ is electrically stimulated and conduction velocities were recorded for central sensory pathways [15]. TMS was applied to the motor cortex and motor evoked potentials (MEPs) were recorded from contralateral extremity muscles [16].

EPs recordings revealed enhanced amplitude of P100 of VEP and delayed N20 (cortical point) of SSEP. TMS recordings revealed increased size of MEPs and short CSP (table 1). The cortical silent period (CSP) is referred to the period of electromyographic suppression elicited when an individual is instructed to maintain muscle contraction and a single suprathreshold TMS pulse is applied to the motor cortex contralateral to the target muscle. CSP is considered as a post-TMS inhibitory phenomenon that reflects the integrity of GABA-ergic intracortical inhibitory control [17].

**Table 1:** Transcranial magnetic stimulation results (before and after treatment)

	Patient		Control
		
	RT	LT	
**MEP Amplitude, **mv			0.94 ± 0.4
*Before treatment*	6.0	4.6	
*After treatment by:*			
15 days	0.82	1.4	
30 days	0.80	0.89	
60 days	0.85	0.80	
90 days	0.82	0.86	

**CSP, ms**			94.8 ± 5.9
***Before treatment***	75	71	
***After treatment by:***			
**15 days**	125	134	
**30 days**	135	132	
**60 days**	132	135	
**90 days**	135	135	

**MEP**: Motor evoked potential, **CSP**: cortical silent period

Valproate (1000 mg/day) was prescribed as a prophylactic treatment. VPA is a known antiepileptic drug which increases brain levels of GABA or enhances GABA action. Occasionally, eletriptan (Relpax 40 mg per needed) was prescribed as a treatment for acute migraine. Triptans constrict blood vessels and possibly inhibit inflammation of the blood vessels (vascular element and not neural). Shortly after the start of treatment, the patient experienced marked clinical and neurophysiological improvement (tables 1 and 2). The decision to continue VPA for at least 6-8 months was discussed with the patient. A gradual and slow decrease in the dose will be undertaken if the patient judges the amelioration of clinical manifestations as sufficient. Tapering of treatment will be undertaken over a period of 6-8 weeks. If there is a clinical aggravation during tapering, the previous (or initial) dose will be repeated. The possibility of life-long treatment with VPA was discussed with the patient due to unusual manifestations and complex pattern of inheritance of his illness.

**Table 2:** Evoked potentials results (before and after treatment)

	Patient		Control
		
	RT	LT	
**VEP, ms**			93.2 ± 3.5
**P100, ms**			
*Before treatment*	98.5	96.2	
*After treatment by:*			
15 days	101.4	103.8	
30 days	102.0	100.5	
60 days	105.5	99.6	
90 days	100.2	101.5	
**Amplitude, μv**			12.5 ± 5.4
*Before treatment*	32.6	43.2	
*After treatment by:*			
15 days	6.5	6.1	
30 days	7.1	6.8	
60 days	6.8	6.5	
90 days	6.2	6.4	

**SSEP, ms**			
**N20 (cortical)**			19.1 ± 0.9
***Before treatment***	20.5	25.2	
***After treatment by:***			
**15 days**	19.35	24.75	
**30 days**	19.50	18.7	
**60 days**	18.73	19.2	
**90 days**	19.10	18.9	

**VEP**: visual evoked potential; **SSEP**: somatosensory evoked potential

Interestingly, as the patient recognized eating fish or its products as a trigger for abdominal colic, he frequently tried fish to evaluate the therapeutic efficacy of VPA.

This study was conducted according to the principles established in Helsinki and approved by Assiut University Hospital ethics committee. Informed written consent was obtained from the patient to publish the details of his clinical history, laboratory and imaging data.

## Discussion

In clinical practice, the presence of family history of common migraine and a trigger for the attack remain the key tools to explain why this patient was born with a liability for migraine. In general, heritability in migraine is estimated to be 40-60% [18]. Migraine phenotypes are genetically complex disorders due to the presence of different liability loci for migrainous headache or aura, variable genetic load (multiple susceptibility genes), and various polymorphisms in the related genes [19]. In families with common migraine, migraine susceptibility genes (genetic load or gene penetrance) can be cumulative. This determines the critical attack threshold which can be modulated by external (e.g. psychosocial stress, preventive therapies ...etc) and/or internal factors (e.g. hormonal status, anxiety ...etc) [20]. Genetic load not only determines the severity of the migrainous disorder, but also it determines complications as chronification by medication overuse [21].

Analyzing the clinical and neurobiological characteristics, the onset of autonomic phenomena, which was manifested as abdominal colic, abdominal flushing, nausea, pallor, diarrhea, tachycardia and fear or anxiety, simulate to a great extent auras of temporal lobe epilepsy (TLE). The amygdale, hypothalamus and medullary cranial nerve nuclei are possible generators of abdominal colic and autonomic manifestations, in support: 1) discharges arising in the amagdala and transmitted to the gut via dense direct projections to the dorsal motor nucleus of the vagus might be responsible for gastrointestinal tract (GIT) symptoms in TLE, 2) sympathetic pathways from the amygdala to the GIT can be activated via the hypothalamus, 3) there are direct sensory pathways from the bowel via the vagus nerve to the solitary nucleus of the medulla which are heavily connected to the amygdala. These pathways can be activated during intestinal contractions, and 4) recently, it has been suggested that orexinegic neurons in the lateral hypothalamus may be a generator for a migraine attack [22].

Additionally, at adolescence, the patient developed manifestations of Alice in Wonderland Syndrome which mainly composed of complex visual illusions. The described distortions of objects and body images in absence of a concomitant attack of vertigo are suggestive of involvement of the posterior part of the right parietal lobe (non-dominant) with and without involvement of the temporal lobe. The occurrence of auditory illusions (consisted of loudening or muffling of ambient sounds) suggests involvement of the posterior superior temporal lobule. While the primary visual hallucinations associated with migraine suggest involvement of the striate and extra-striate regions of the occipital lobe [9]. Patients with AIWS know that the distorted experiences of their bodies or the environment are not real. The complex perceptual changes in AWLS are mainly visual but not hallucinatory. In, AWLS, the sensory inputs are normal but the faulty sensory processing results in an inappropriate pattern of cortical excitation, while in hallucination, the sensory inputs or hallucinations represent a release phenomena due to defective sensory input caused by the faulty stimulation of the associative cortices [12,13].

The presence of atypical manifestations, progressive weight loss and reduced growth mandate medical diagnostic workup for exclusion of an organic cause. Neuroimaging and neurophysiologic assessments were needed to exclude other causes of AWLS as epilepsy [9], infectious mononucleosis [23], epstein-Barr virus infection [24], cerebral vasculitis [25], psychosis [26] and drug adverse effect or intoxication [27,28]. Normal diffusion-weighted MRI is consistent with the diagnosis of migraine. Changes in cerebral blood flow were identified during and in-between headaches in migraine with and without aura. White matter hyperintensities were found in MRI of patients with migraine [29]. Recently, cerebral perfusion abnormalities were identified by functional neuroimaging studies (as SPECT) done during or immediately after attacks of migraine with aura [30].

SEPs and TMS results indicate cortical hyperexcitability in multiple brain regions or diffuse brain involvement. Enhanced amplitude of P100 of VEP is indicative of occipital cortical hyperexcitability. Delayed N20 (cortical point) of SSEP is indicative of parietal lobe dysfunction. Increase in the size of motor responses or MEPs and short CSP are indicative of motor neuronal hyperexcitability. In accordance, independent evidences from VEPs and TMS, recordings and psychophysics indicate the presence of deficient habituation and changes in neuronal excitability during and in between the attacks of migraine [31,32].

The neurophysiologic changes observed in migraine could offer explanation for the migraine mechanism. In general, cortical hyperexcitability is due to imbalance between the cortical inhibitory GABA-ergic (gamma aminobutyric acid) and excitatory glutaminergic circuits [33]. VPA increases brain levels of GABA or enhances GABA action, thus improves the patient's symptoms. Under normal conditions, cortical excitability is largely regulated by GABA-ergic inhibitory interneurons through GABA receptors (type A and B). GABA receptors occupy ~70-80% of the 2^nd^, 3^rd ^and 5^th ^cortical layers. Fast and early cortical motor inhibitions are mediated by GABA_A _receptors, while slow and late G-protein-coupled inhibitions are mediated by GABA_B _receptors. In fact, neuronal excitability is governed by the complex interplay of channel currents. Changes in ion channels as a result of hereditary mutations could result in minimal or episodic changes in neuronal micro-environment. These changes might be sufficient to cause transient neuronal hyperexcitability. It has been found that the subpopulations of cortical neurons have different physiological properties in response to different stimuli, thus might be differentially modulated in migraine extremes. Furthermore, expression of a single mutation in different cell types, networks and developmental periods has been suggested to underlie the coexistence of common migraine, atypical migraine auras, or a non-epileptic paroxysmal disorder in one individual or family [34]. These explanations might concur with the manifestations observed in our patient.

## Conclusions

We suggest that the occurrence of two different migraine phenomena in one patient might be the result of chronicity and the concomitant central sensitization process [35]. Due to the complex manifestations and unusual pattern of inheritance, it is important to point that the treatment option of this patient with VPA may be life-long.

## Competing interests

The authors declare that they have no competing interests.

## Pre-publication history

The pre-publication history for this paper can be accessed here:

http://www.biomedcentral.com/1471-2377/10/2/prepub
